# HPV-related oropharyngeal cancer prevalence in a middle eastern population using E6/E7 PCR

**DOI:** 10.1186/s13027-019-0268-z

**Published:** 2020-01-06

**Authors:** Christopher A. Maroun, Karine Al Feghali, Henri Traboulsi, Helene Dabbous, Fatmeh Abbas, Gabriel Dunya, Georges Ziade, Rami Mahfouz, Bassem Youssef, Hani Tamim, Fady Geara, Ibrahim Khalifeh, Roger V. Moukarbel

**Affiliations:** 1 Department of Otolaryngology Head and Neck Surgery, American University of Beirut Medical Center, 6th Floor, Hamra, Beirut, 1107 2020 Lebanon; 2 Department of Radiation Oncology, American University of Beirut Medical Center, Beirut, Lebanon; 3 Department of Pathology and Laboratory Medicine, American University of Beirut Medical Center, 2nd Floor, Hamra, Beirut, 1107 2020 Lebanon; 4 Biostatistics Unit, Clinical Research Institute, Faculty of Medicine, American University of Beirut, Beirut, Lebanon

**Keywords:** Oropharyngeal cancer, Head and neck squamous cell carcinoma, Human papilloma virus, Middle East, Lebanon

## Abstract

**Background:**

Given the paucity of data and widely variable rates that have been reported, the main objective of this study was to examine the prevalence of HPV-positivity in oropharyngeal squamous cell carcinoma (OPSCC) in Middle Eastern patients presenting to one of the region’s largest tertiary care centers using polymerase chain reaction (PCR) amplification of the HPV E6/E7 oncogenes, a highly sensitive and specific method of detection.

**Methods:**

Medical charts and archived pathological specimens were obtained for patients diagnosed with biopsy proven oropharyngeal cancer who presented to the American University of Beirut Medical Center between 1972 and 2017. DNA was extracted from paraffin-embedded specimens and tested for 30 high-risk and low-risk papilloma viruses using the PCR-based EUROarray HPV kit (EuroImmun).

**Results:**

A total of 57 patients with oropharyngeal cancer were initially identified; only 34 met inclusion/exclusion criteria and were included in the present study. Most patients were males (73.5%) from Lebanon (79.4%). The most common primary tumor site was in the base of tongue (50%), followed by the tonsil (41.2%). The majority of patients (85.3%) tested positive for HPV DNA.

**Conclusion:**

The prevalence of HPV-positivity amongst Middle Eastern OPSCC patients, specifically those from Lebanon, may be far greater than previously thought. The Lebanese population and other neighboring Middle Eastern countries may require a more vigilant approach towards HPV detection and awareness. On an international level, further research is required to better elucidate non-classical mechanisms of HPV exposure and transmission.

## Introduction

Human papillomavirus (HPV) is well-established as a distinct etiological risk factor for oropharyngeal cancer [1]. Several studies in the United States have demonstrated a noticeable increase in the incidence of oropharyngeal squamous cell carcinoma (OPSCC) in the last few decades that has been largely attributed to the carcinogenic potential of oral HPV infection [2–5]. Notably, the increasingly important role of HPV infection in the development of OPSCC has led to a dramatic shift in the profile of OPSCC patients seen.

Patients with HPV-related OPSCC are reportedly more likely to be younger, white, male, married, and college-educated than their HPV-negative counterparts [6]. Additionally, HPV-related OPSCC has been significantly less associated with classical oncologic risk factors such as tobacco smoking and alcohol, and more so with exposure to marijuana and sexual activity, including increasing number of oral sexual partners [6]. Clinically, HPV-related OPSCC is more likely to arise from tonsillar tissue compared to other subsites of the oropharynx, which attests to its infectious etiology [5]. Interestingly, HPV-related OPSCC is also more likely to present at an advanced stage, with significant nodal disease despite small primary tumors; regardless, its prognosis tends to be more favorable [7, 8]. All of the previously mentioned characteristics demonstrate that HPV-related OPSCC is clearly a unique entity both clinically and demographically. Although, reports examining HPV-related OPSCC more recently have suggested that its incidence has been increasing even among older populations, who also display an attenuated survival benefit compared with younger patients [9]. This highlights the importance of HPV infection even further, as its implications may no longer be isolated to specific populations, as previously thought.

The prevalence of HPV-related OPSCC appears to differ widely by region, potentially owing to differences in social practices attributed to different cultures around the world. Data regarding the prevalence of HPV-related OPSCC in the Middle East region in particular is scarce. Given the paucity of data and widely variable rates that have been reported, the main objective of this study was to examine the prevalence of HPV-positivity in OPSCC in Middle Eastern patients presenting to one of the region’s largest tertiary care centers using polymerase chain reaction (PCR) amplification of the HPV E6/E7 oncogenes, a highly sensitive and specific method of detection. Secondary outcomes included investigating the impact of HPV tumor status on prognosis in this cohort, specifically overall survival and recurrence-free survival.

## Materials and methods

This study was reviewed and approved by the Institutional Review Board at the American University of Beirut (AUB) in Beirut, Lebanon.

### Participant recruitment

Patients diagnosed with biopsy proven oropharyngeal cancer who presented to our institution between 1972 and 2017 were identified according to the *International Classification of Diseases 9 (ICD-9)* codes 141.0 *Malignant neoplasm of base of tongue* and 146.x *Malignant neoplasm of oropharynx* (including all subsites). Medical charts were retrieved from the medical records department and archived pathological specimens were obtained from the pathology department when available. Verbal consent was obtained over the telephone from living patients. No consent was obtained for the inclusion of deceased patients as this was considered non-human subject research.

### Inclusion criteria

Patients of all ages and both genders with the following criteria were included: primary lesions of the oropharynx and its subsites, specifically the palatine tonsils, base of tongue, soft palate, posterior oropharyngeal wall, anterior pillars, posterior pillars, and valleculae; primary lesions of the oropharynx with extension to neighboring/distant sites or metastasis; patients originating from Middle Eastern countries.

### Exclusion criteria

The following were considered as exclusion criteria: patients with metastatic disease from distant sites to the head and neck; patients with non-oropharyngeal head and neck cancers; patients with non-squamous cell tumors; patients who did not have any records or pathological specimens available at our institution.

### Data collection

A retrospective chart review was performed whereby demographic and clinical data were obtained regarding age at diagnosis, gender, country of origin, history of tobacco and alcohol use, site of primary tumor, TNM classification, stage at presentation, and modality of treatment including surgery (resection +/− neck dissection), radiation therapy and systemic therapy (chemotherapy or cetuximab). Tobacco use was defined as a smoking history of ten pack years or greater, where one pack is equivalent to smoking 20 cigarettes, and a pack year is defined as the number of packs smoked per day multiplied by the number of years the person has smoked. TNM classification and staging information were retrieved directly from medical charts or inferred from clinical records and imaging reports according to the American Joint Committee on Cancer (AJCC) Cancer Staging Manual 7th edition. Survival data was obtained, including status at last available follow-up regarding recurrence and death, up until five years from diagnosis. No patient identifiers were collected. Pathological specimens, including paraffin-embedded biopsy and surgical tissues, and cryopreserved fine-needle aspirates, were retrieved, coded and de-identified.

### Human papillomavirus testing

DNA was extracted from all samples using Qiagen reagents. DNA aliquot was then tested using EUROarray HPV kit (EuroImmun). The test is designed for molecular diagnostic in vitro detection and typing of 30 human anogenital high-risk and low-risk papilloma viruses (HPV 6, 11, 16, 18, 26, 31, 33, 35, 39, 40, 42, 43, 44, 45, 51, 52, 53, 54, 56, 58, 59, 61, 66, 68, 70, 72, 73, 81, 82, 89) from DNA preparations. The test system is based on detection of the viral oncogenes E6/E7. In the first reaction step, regions of the viral oncogenes E6 and E7 from HPV that are present in the sample are amplified and fluorescently labelled by means of PCR using a multiplex primer system. In the second reaction step the products are detected using an oligonucleotide microarray. The specific binding (hybridization) of a fluorescing PCR product to the corresponding oligonucleotide probe is detected using a special Microarray Scanner (EUROIMMUN). The EUROArrayScan (EuroImmun) software evaluates all spot signals and generates the test results. An additional primer system that amplifies a region of human genomic DNA is integrated into the test system. This serves as a positive control for the DNA preparation. Correctly taken smears will contain cervical cells and hence their genomic DNA.

### Data analysis

Associations between HPV status, and demographic and clinical characteristics were assessed using the Mann-Whitney U Test for continuous variables and Fisher’s exact test for categorical variables. A two-sided *p*-value less than 0.05 was considered statistically significant. Kaplan-Meier analysis with the Log rank test was used to conduct unadjusted survival analysis for overall and recurrence-free survival with time-to-outcome calculated starting at the date of diagnosis. Censoring for subjects with no reportable events was performed at date of last follow-up. All statistical analyses were performed using SPSS Statistics for Windows Version 25.0 (IBM Corp., Armonk, NY).

## Results

A total of 57 patients with oropharyngeal cancer was initially identified; only 34 met the above cited criteria and were included in the present study. Demographic and clinical characteristics for all patients were stratified by HPV status and are summarized in Table 1. Overall mean age at diagnosis was 58.9 ± 9.3 years. Most patients were males (73.5%) from Lebanon (79.4%). The remaining patients originated from Syria, Jordan, Iraq, and Palestinian territories. A majority of patients reported a history of tobacco use (70.6%) and approximately half of patients reported a history of alcohol (47.1%), with half of those reporting daily use (8/16, 50%). The most common primary tumor site was in the base of tongue (50%), followed by the tonsil (41.2%). Only 3 tumors originated from the soft palate. Most patients presented with stage IV disease (79.4%), however only 6 patients presented with distant metastasis. Only 4 patients had a negative lymph node examination clinically or on imaging at the time of evaluation. Treatment information was missing from 4 patients. Of those with treatment information available, 8 patients underwent single-modality treatment, 15 underwent dual-modality treatment, and 7 underwent tri-modality treatment.

**Table 1:** Demographic and clinical characteristics of patients with oropharyngeal squamous cell carcinoma presenting to the American University of Beirut Medical Center from 1972 to 2017

Descriptive	Overall *N* = 34	HPV Negative *N* = 5	HPV Positive *N* = 29	*P*-value
Age at diagnosis in years, mean(sd)	58.9 (9.3)	57.6 (7.8)	59.1 (9.6)	0.645
Male, *n*(%)	25 (73.5)	3 (60.0)	22 (75.9)	0.591
Country, *n*(%)ss				1.000
Lebanon	27 (79.4)	5 (100)	22 (75.9)	
Syria	3 (8.8)	0 (0)	3 (10.3)	
Palestinian Territories	1 (2.9)	0 (0)	1 (3.4)	
Jordan	1 (2.9)	0 (0)	1 (3.4)	
Iraq	2 (5.9)	0 (0)	2 (6.9)	
Tobacco use, *n*(%)	24 (75.0)	3 (75.0)	21 (75.0)	1.000
Any alcohol use, *n*(%)	16 (50.0)	2 (50.0)	14 (50.0)	1.000
Social	7 (46.7)	1 (50.0)	6 (46.2)	
Daily	8 (53.3)	1 (50.0)	7 (53.8)	
Primary site, *n*(%)				1.000
Tonsil	14 (41.2)	2 (40.0)	12 (41.4)	
BOT	17 (50.0)	3 (60.0)	14 (48.3)	
Soft Palate	3 (8.8)	0 (0)	3 (10.3)	
Stage at presentation, *n*(%)				0.660
I	1 (3.0)	0 (0)	1 (3.6)	
II	1 (3.0)	0 (0)	1 (3.6)	
III	4 (12.1)	1 (20.0)	3 (10.7)	
IV	27 (81.8)	4 (80.0)	23 (82.1)	
Clinical T stage, *n*(%)				1.000
TX	3 (10.0)	0 (0)	3 (11.5)	
T1	3 (10.0)	0 (0)	3 (11.5)	
T2	9 (30.0)	1 (25.0)	8 (30.8)	
T3	6 (20.0)	1 (25.0)	5 (19.2)	
T4	9 (30.0)	2 (50.0)	7 (26.9)	
Clinical N stage, *n*(%)				0.121
NX	1 (3.2)	1 (25.0)	0 (0)	
N0	4 (12.9)	0 (0)	4 (14.8)	
N1	4 (12.9)	1 (25.0)	3 (11.1)	
N2	22 (71.0)	2 (50.0)	20 (74.1)	
N3	0 (0)	0 (0)	0 (0)	
M stage, *n*(%)				0.359
MX	1 (3.1)	0 (0)	1 (3.7)	
M0	25 (78.1)	3 (60.0)	22 (81.5)	
M1	6 (18.8)	2 (40.0)	4 (14.8)	
Surgery only, *n*(%)	5 (14.7)	0 (0)	5 (17.2)	
Surgery + Radiation, *n*(%)	1 (2.9)	1 (20)	0 (0)	
Surgery + Radiation + Systemic therapy, *n*(%)	7 (20.6)	1 (20)	6 (20.7)	
Radiation only, *n*(%)	2 (5.9)	0 (0)	2 (6.9)	
Radiation + Systemic therapy, *n*(%)	14 (41.2)	2 (40)	12 (41.4)	
Systemic therapy only, *n*(%)	1 (2.9)	1 (20)	0 (0)	

*N,n* count, *sd* standard deviation; Information was missing from 1 patient for Stage, 7 patients for T stage, 3 patients for N stage, 5 patients for M stage, and 4 patients for treatment

The majority of patients (29/34, 85.3%) tested positive for HPV DNA. There were no statistically significant differences regarding any demographic or clinical variables when comparing HPV positive and HPV negative groups. In particular, all 5 HPV negative patients originated from Lebanon. Out of 8 nonsmokers, 7 were HPV positive. The majority of HPV positive patients presented with advanced nodal disease (N2, 69%). Only 4 HPV positive patients presented with distant metastasis. Interestingly, all 3 soft palate tumors tested positive for HPV DNA.

**Fig. 1:**
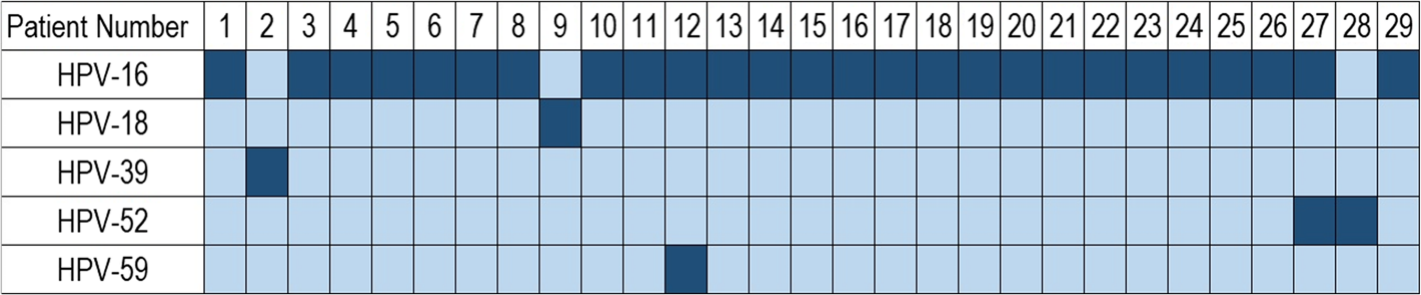
Visual summary of subtypes of HPV detected by polymerase chain reaction (*n* = 29). Dark color represents positivity. The most common subtype was HPV-16 (26/29, 89.7%). The remaining patients (3/29, 10.3%) tested positive for either HPV-18, HPV-39, or HPV-52 alone. Among the HPV-16 positive patients, one patient tested positive for HPV-52, and another was positive for HPV-59

Figure 1 provides a visual summary of the HPV subtypes that were found positive in each patient sample. The most common subtype was HPV-16 (26/29, 89.7%). The remaining patients (3/29, 10.3%) tested positive for either HPV-18, HPV-39, or HPV-52 alone. To note, among the HPV-16 positive patients, one patient tested positive for HPV-52, and another was positive for HPV-59.

Overall and recurrence-free survival data were available for 24 and 23 patients, respectively. There was no significant difference in overall (Log rank *p* = 0.125) or recurrence-free survival (Log rank *p* = 0.941) between HPV negative and HPV positive groups. Mean overall survival for the HPV positive group was 56.5 ± 3.4 months, and mean recurrence-free survival was 42.8 ± 5.4 months.

## Discussion

The prevalence of HPV-related OPSCC in our study sample was found to be 85.3%, which is comparable to the higher rates reported in many developed countries [2–5, 10]. This result was unexpected, given that HPV-related OPSCC is classically associated with sexual activity, and most Middle Eastern countries are historically more conservative [6]. However, the patient profile of our study sample generally fits that of an HPV-related patient as described in the literature [6–8]. Most HPV-related patients in our study were males who presented with nodal metastases and advanced stage at diagnosis. Additionally, the most common sites affected in our study sample were the base of tongue, followed closely by the tonsils. Both are sites of lymphoid tissue, which are known to be more susceptible to HPV infection and related oncogenesis [1]. Of note, all three soft palate tumors were HPV-related. The prevalence of HPV-related OPSCC located in the soft palate has not been well studied, however rates of 0–67% have been reported in the literature [11]. While it is established that HPV-positivity in the soft palate is generally lower than in other subsites, concomitant tobacco smoking likely increased those patients’ risk of OPSCC. Despite the high prevalence of HPV-positivity, a majority of the patients in our study were smokers, with approximately half reporting a history of alcohol use.

While the largest number of studies on HPV-related OPSCC may have been conducted in North America and Europe, there is a multitude of studies performed around the world that echoes the importance of HPV in the development of OPSCC. A meta-analysis examining HPV-related OPSCC trends by region over time between 1970 and 2008 found that rates in North America increased from 50.7 to 69.7%, and those in Europe increased from 35.3 to 73.1% [10]. One study conducted in Australia found that in the period from 2006 to 2010, 63.5% of OPSCC patients tested positive for HPV by PCR and p16 immunohistochemistry (IHC) [12]. In contrast, a study conducted in Malaysia found that in the period from 2004 to 2015, only 25% of OPSCC patients tested positive for HPV by p16 IHC [13]. Our data is discordant with another study conducted in Lebanon on 30 patients with OPSCC treated between 2010 and 2016, who found an HPV prevalence of 27% by PCR [14]. A Turkish study on 81 patients found that the prevalence of HPV-related OPSCC increased from 38 to 64% between the periods of 1996–2003 and 2004–2011 [15].

Oral-genital and oral-oral contact has been implicated in the transmission of oral HPV infection and subsequent development of OPSCC [16]. There is also evidence that the sharing of marijuana cigarettes may play a role in the transmission of HPV [17]. It has been speculated that lower rates of HPV-related OPSCC previously reported in the Middle East may be due to differences in sexual practices compared to Western countries [15]. Interestingly, there have been several studies that report various non-sexual methods of HPV transmission. One systematic review found a prevalence of HPV DNA in virgins ranging between 0 and 50%, as reported in the literature [18]. Multiple studies have found that gynecological equipment including gloves, endovaginal ultrasound probes, colposcopes, and specula may be contaminated with HPV DNA even after routine cleaning [18, 19]. Additionally, another study conducted in Tanzania detected HPV DNA in fingertip, oral and bathroom samples from unvaccinated adolescent girls [20]. Projecting from these data, it can be speculated that, in the Middle East in particular, cultural practices that involve fomite sharing such as hookah smoking and mate drinking may contribute to the non-sexual transmission of oral HPV, thus providing a potential explanation for HPV transmission in addition to underreported sexual practices.

There are over 200 subtypes of HPV that have been identified. The carcinogenic potential of high risk subtypes such as HPV-16, the most commonly isolated subtype from the oropharynx, was first established in cervical cancer [21]. Other high risk subtypes that have been reported include HPV-18, 31, 33, 35, 39, 45, 51, 52, 56, 58, 59, 66 and 68 [1]. Oncogenesis is primarily driven by two viral proteins that disrupt critical pathways involved in tumor suppression: E6, which binds to the tumor suppressor protein p53, and E7, which interacts with a number of host-proteins, most notably of the pRb family [1, 21]. The collective disruption of these pathways leads to a bypass of cellular checkpoints in the presence of DNA damage, leading to genomic instability and eventually malignant progression [22]. A hallmark downstream effect of these HPV related oncoproteins is the accumulation of p16 protein within cells as a result of CDKN2A overexpression [1].

Multiple methods for HPV detection in patient tumor samples have been described and are utilized in daily practice. The most prevalent method to date, owing to its cost-effectiveness, ease of interpretation, and high sensitivity, is IHC for p16 as a surrogate marker of HPV positivity [23]. However, as there are other mechanisms through which p16 may be overexpressed, p16 has its limitations in terms of specificity [24]. In fact, one study by Singhi and Westra found that 16% of tumors that were negative for HPV DNA by in situ hybridization (ISH) tested positively for p16 overexpression [25]. In situ hybridization for HPV-16 DNA is another widely used method; however, its sensitivity is limited since other HPV subtypes are disregarded [24]. The gold standard for detecting transcriptionally active HPV infection is considered by many to be detection of HPV E6 and E7 mRNA transcripts using polymerase chain reaction (PCR) [23]. However, PCR for HPV DNA provides higher sensitivity for detection, especially amongst older samples, and allows for wider genotypic characterization across HPV subtypes [26]. While we did not utilize p16 assays as a method of HPV detection in our study, our use of PCR for E6/E7 provides the highest level of confidence in our results.

As most of the patients in our study are Lebanese, the potential implications of these findings most strongly apply to the Lebanese population. An important aspect of our study will be its impact on health education and on raising awareness about HPV-related OPSCC, which could play a role in controlling the increasing prevalence of this entity and promoting vaccination among young adults. With the advent of vaccines for prevention of HPV-related cervical cancer, the incidence of HPV-related OPSCC might decrease with the decrease of HPV-positivity and transmission as a whole [8]. However, a recent study conducted in Lebanon found an HPV vaccination uptake rate of 2.5% and a rate of awareness about HPV infection of 34% amongst mothers of schoolgirls in their sample [27]. These data are alarming, and indicate that there is a clear need for action on multiple levels in order to even begin the prevention of HPV transmission and associated diseases, including OPSCC.

Our study has some limitations, primarily owing to its retrospective nature. Because of the relatively low incidence of OPSCC in our population in general, we were required to obtain medical records spanning a somewhat large timeframe. As a result, the quality of the records obtained differed greatly depending on when the record was originally created. We were not able to obtain adequate survival data to completely analyze our secondary outcomes, due to the low number of HPV-negative patients and the high number of censored subjects. Additionally, we were only able to include patients who had either a biopsy or resected specimen available at our institution. Due to our utilization of a DNA based PCR detection method, we are not able to make definitive conclusions regarding genomic integration or disease association. However, the prevalence of high-risk HPV subtypes amongst our OPSCC specimens itself is impressive. Finally, as OPSCC is uncommon in our country, our sample size was low; however, this will be the subject of future studies.

## Conclusion

We have demonstrated that the prevalence of HPV-positivity amongst Middle Eastern OPSCC patients, specifically those from Lebanon, may be far greater than previously thought. The implications of this are many, as HPV-related OPSCC behaves much differently than HPV-negative OPSCC both clinically and prognostically, and often may benefit from a different approach to treatment. The implications on a national level are that the Lebanese population and other neighboring Middle Eastern countries may require a more vigilant approach towards HPV detection and awareness, starting from the earliest stages of possible exposure. On an international level, our findings indicate that further research is required to better elucidate non-classical mechanisms of HPV exposure and transmission, and that HPV-positivity in OPSCC may play an important role regardless of geographic location.

## Data Availability

The dataset used during the current study is available from the corresponding author on reasonable request.

## Abbreviations

AJCC: American Joint Commission on Cancer
AUB: American University of Beirut
HPV: human papillomavirus
ICD: International Classification of Diseases
IHC: immunohistochemistry
ISH: in-situ hybridization
OPSCC: oropharyngeal squamous cell carcinoma
PCR: polymerase chain reaction
